# Digital interventions for alcohol use and alcohol use disorders in low- and-middle-income countries: a systematic review

**DOI:** 10.1093/oodh/oqaf004

**Published:** 2025-01-12

**Authors:** Payal Khatore, Hizkia Yolanda, Jaeden Joyner, Abhijit Nadkarni

**Affiliations:** Centre for Global Mental Health, London School of Hygiene and Tropical Medicine, Keppel Street, London WC1E 7HT, UK; Centre for Global Mental Health, London School of Hygiene and Tropical Medicine, Keppel Street, London WC1E 7HT, UK; Centre for Global Mental Health, London School of Hygiene and Tropical Medicine, Keppel Street, London WC1E 7HT, UK; Centre for Global Mental Health, London School of Hygiene and Tropical Medicine, Keppel Street, London WC1E 7HT, UK; Department of Population Health, London School of Hygiene and Tropical Medicine, Keppel Street, London WC1E 7HT, UK; Addictions and Related Research Group, Sangath, Porvorim, Goa 403501 India

**Keywords:** digital interventions, alcohol use disorders, low-and-middle-income countries, substance use, alcohol use, systematic review

## Abstract

**Background:**

Despite the high burden of alcohol use and alcohol use disorders (AUDs) in low-and-middle-income countries (LMICs), access to health care is poor. Digital interventions (DIs) have recently emerged as promising avenues for addressing substance use. Such interventions could potentially address barriers to help-seeking in LMICs, such as travel costs, shortage of professionals, stigma, etc.

**Aim:**

To synthesize evidence on the effectiveness and implementation of DIs for AUDs in LMICs.

**Methods:**

The systematic review had a comprehensive search strategy that combined search terms for DIs (e.g. SMS, eHealth), alcohol use (e.g. hazardous drinking) and LMICs (e.g. India). Studies presenting primary data that reported effectiveness (e.g. relapse) and/or implementation or intervention-related outcomes (e.g. feasibility) of DIs for AUDs in LMICs were eligible. Three databases (EMBASE, MEDLINE and PsycINFO) were searched from their inception till June 2023. Data was extracted in relevant categories and analysed.

**Results:**

Twenty-one reports from 19 studies were included. Types of DIs ranged from standalone mobile applications and web portals to human-delivered interventions via digital platforms. 12 studies reported positive or partially positive alcohol use outcomes (e.g. number of drinking days, abstinence). DIs with human involvement were found to be more effective than standalone DIs. Additionally, high levels of acceptability, feasibility and satisfaction were reported across interventions.

**Conclusion:**

DIs are acceptable and feasible in LMICs and broadly effective in improving alcohol use outcomes. Firm conclusions could not be drawn because of methodological issues such as small sample sizes, short follow-up periods and limited generalisability. Adequate investment, improved research methodology and increased focus on implementation outcomes are required for determining the role that DIs can play in addressing AUDs in LMICs.

## INTRODUCTION

Alcohol use disorders (AUDs) are the most prevalent of all substance use disorders (SUDs), with approximately 100 million cases globally in 2016 [1]. Low-and-middle-income countries (LMICs) account for more than 85% of alcohol-related deaths globally [2]. In 2018, Russia, Ukraine, Belarus, Lesotho and Burundi were amongst the countries with the highest alcohol-attributable disability-adjusted life years (DALYs) per 100 000 people [1]. However, only a minority across LMICs have access to adequate treatment for AUDs; for example, the treatment gap is as high as 80–90% in countries such as Mexico and Turkey [3]. This is due to workforce shortages, overburdened health systems [4], limited access to evidence-based treatments, minimal help-seeking because of stigma, poor policy implementation and inadequate political support [3]. Given the increasing and widespread availability of alcohol, it is imperative to use public health interventions to address the harmful consumption of alcohol [2].

A digital intervention (DI) is defined as a ‘discrete functionality of the digital technology to achieve health sector objectives’ [5]. DIs have recently emerged as promising avenues for addressing a variety of health issues [e.g. cardiovascular disease, human immunodeficiency virus (HIV)] [6, 7], including mental health problems and SUDs [8–11]. DIs can be instrumental in addressing the treatment gap for AUDs in LMICs by reaching remote populations that lack access to clinical services, connecting people across borders for support, and, in turn, empowering them to seek help [3].

In high-income countries (HICs), alcohol-focused reviews of DIs have found stronger evidence compared to the broader evidence for SUDs [12, 13]. A majority of remotely delivered alcohol interventions showed encouraging results for improving alcohol use outcomes [14], and a Cochrane review on community-based personalized DIs found moderate quality evidence that DIs are potentially better than minimal or no intervention for reducing hazardous alcohol consumption [15].

In 2019, the World Health Organization (WHO) urged stakeholders to generate meaningful evidence for the effectiveness and usability of digital technologies in order to ensure that investment in them does not divert limited resources from non-DIs [16]. This is especially relevant for LMICs that have scarce resources but also rapid access to technological advancements. As of 2022, the percentage of the population with mobile phone subscriptions in low-income, lower-middle-income and upper-middle-income countries was 49%, 65% and 76%, respectively [17]. Moreover, for the first time, 50% of the population in LMICs had access to mobile internet in 2021 [18]. Despite this rapid growth, there is a large discrepancy in the state of digital health in LMICs compared to HICs. DIs in LMICs can look very different from those in HICs and may have to rely on relatively low-end technology. Despite that, most DIs are not tailored to local contexts but simply imported from the West [19].

A broad systematic review on the effectiveness of digital psychological interventions for mental health problems in LMICs suggested that future research should evaluate the evidence for DIs related to specific mental health problems in order to draw meaningful conclusions for future research [20]. However, there have been no dedicated attempts to systematically synthesize evidence on DIs for AUDs in LMICs. The existing reviews have narrowly focused on a single type of DI (telehealth interventions for SUDs in LMICs) [21] or examined DIs as a subset of other interventions [DIs as a subset of brief interventions (BIs) addressing AUDs] [22].

Therefore, our comprehensive systematic review aims to plug that gap by summarizing the current state of evidence on DIs for risky alcohol use and AUDs in LMICs. More specifically, our objectives are to (i) examine and synthesize the effectiveness of DIs in preventing and treating risky alcohol use and AUDs, (ii) describe the content, design and delivery of the interventions in terms of core components and implementation processes (e.g. fidelity, uptake, adherence, etc.) and (iii) synthesize information about the acceptability and feasibility of various DIs in their respective contexts.

## MATERIALS & METHODS

Our review complies with the Preferred Reporting Items for Systematic Review and Meta-analysis (PRISMA) guidelines (Appendix 1). The protocol for our review was originally registered a priori on PROSPERO on 26 June 2023 (registration number CRD42023439120) with an amendment on 19 July 2023 to correct a typing error.

### Design

Systematic review.

### Eligibility criteria

Studies that involved participants with hazardous, risky, harmful or dependent drinking behaviours or AUDs with or without coexisting physical or mental health conditions were included, irrespective of age and gender. The type of alcohol use must be defined either clinically using diagnostic manuals like the Diagnostic and Statistical Manual of Mental Disorders (DSM) or International Classification of Diseases (ICD) or through standardized screening or diagnostic tools.

DI was defined as the use of any digital technology with the stated aim of addressing alcohol use/disorder. This could be standalone i.e. not require human involvement (e.g. text messages, smartphone applications), directly delivered to the potential beneficiaries (individual/group), or be delivered by humans using technology (e.g. telehealth services).

DIs as universal interventions aimed at primary prevention in the general population were excluded. Health systems interventions (e.g. medical records digitization) and interventions that involved no end-user interaction with the technology (e.g. cash transfers) were excluded.

If the DI was not directly aimed at changing alcohol use but the study measured alcohol use outcomes, it was excluded. Studies where digital tools are used only for measurement, screening or follow-up and not as interventions were also excluded. Finally, studies solely exploring delivery agents’ experiences of delivering DIs were also excluded.

For effectiveness studies, eligible comparators were people who did not receive any intervention (inactive control) or those who received any other intervention (active control), whether digital or non-digital (psychological, pharmacological).

Studies were included if they reported (i) outcomes related to alcohol use (e.g. frequency, severity, quantity of alcohol use, abstinence, remission, relapse, recovery) measured using validated measures, (ii) implementation outcomes related to the intervention included uptake, acceptability, feasibility, safety, user engagement, etc., and (iii) outcomes secondarily related to alcohol use/disorders (violence, accidents, adverse events like suicide attempts and hospitalization due to alcohol-related reasons).

Both quantitative and qualitative studies presenting primary data, including experimental studies (e.g. individual RCTs, cluster randomized trials, pragmatic trial), quasi-experimental studies (e.g. controlled before and after designs and interrupted time series design), and non-experimental studies (e.g. cohort studies, repeat cross-sectional studies, single group pre-post-test, post-programme and qualitative studies) were included.

Secondary analyses, reviews, meta-analyses, commentaries, opinion pieces and case series were excluded.

LMICs were defined based on the World Bank categorization (2021), and studies conducted across multiple countries were included if they reported segregated outcomes for one or more LMICs.

We had no restrictions on the year of publication and excluded grey literature.

### Procedures

The search strategy (Appendix 2) was divided into three ‘search concepts’ – digital/technology-based interventions (e.g. mHealth, telemedicine), alcohol use/disorder(s) [e.g. alcoholism, binge drinking (BD)] and LMICs (e.g. Afghanistan, Brazil, developing economy).

We used OVID to search EMBASE, MEDLINE and PsycINFO from each database’s respective dates of establishment [23] till 22 June 2023.

Finally, citation chaining was conducted using Google Scholar [24] for backward and forward citation searching (Backward searching refers to looking at bibliographies of reports and identifying any relevant articles cited by them. Forward searching involves looking at which papers have subsequently cited the report of interest [24]) using each record included in the review.

Search results were imported into Rayyan, a web tool used for collaborative screening and selection [25]. Deduplication using Rayyan was subsequently supplemented through manual deduplication by the primary reviewer (PK). 10% of the titles and abstracts were screened independently by two reviewers (PK, HY) and the rest were screened by only the PK. For the double-screened titles and abstracts, any disagreements were resolved through discussion or through consultation with a third reviewer (JJ). The next step was full-text screening by both reviewers (PK, HY), with disagreements being resolved through discussion or through consultation with a third reviewer (JJ). Concordance scores were calculated for both screening stages by dividing the number of agreements by the total number of records screened by both reviewers.

Data was extracted in the following categories using an electronic data extraction sheet: background (e.g. country), population characteristics (e.g. age), study characteristics (e.g. sample size), intervention and control characteristics (e.g. content), outcome characteristics for effectiveness (e.g. severity) and interventions (e.g. fidelity). The cross-check method [24] was employed wherein the PK extracted the data, and the second reviewer (HY) double-checked it for any errors or missing information.

### Quality appraisal

The quality and risk of bias of each eligible study were assessed by two reviewers using the relevant Joanna Briggs Institute (JBI) critical appraisal tool [26–28]. The Mixed Methods Appraisal Tool (MMAT) was used for mixed-methods studies [29]. An overall quality appraisal score for each study was provided as a measure of the quality. This was calculated by assigning numerical values to the answers for each question (Yes = 1; No = 0; Unclear = no score) and adding the values to arrive at a score that was divided by the total number of questions. This was converted into a percentage such that ≤49% was high, 50–69% was moderate and above 70% indicated a low risk of bias [30]. Disagreements were resolved through discussion to arrive at consensus ratings for each question.

### Data synthesis

Given the heterogeneity in study designs, outcomes and interventions, a narrative synthesis approach was used to analyse the data, following the guidance by Popay *et al*. (2006) [31]. A preliminary synthesis identified patterns in the size and direction of effects and implementation outcomes. This involved organizing data using textual descriptions and tables and grouping it in various clusters by the type of intervention, effectiveness, risk of bias, etc. Heterogeneity between studies was explored through conceptual models and qualitative descriptions to investigate the characteristics of studies and interventions that contributed to various outcomes. The final data groupings were determined based on this and findings were presented as tables consisting of characteristics of studies and interventions, along with outcomes. These were supplemented by qualitative descriptions exploring relationships across tables to provide a comprehensive synthesis of the findings.

## RESULTS

### Search results (Fig. 1)

A total of 9977 articles were identified through the database search, and after deduplication, 8189 records were screened. Of these, 78 reports were eligible for full-text screening. There was 98.8% concordance for the 10% of articles that were screened by both reviewers. After full-text screening, 19 reports met the eligibility criteria for inclusion in the review. There was 82.1% concordance between the two screeners for the full-text screening. Citation chaining yielded two additional reports [32, 33],, and a total of 21 reports were included in the review.

**Figure 1:**
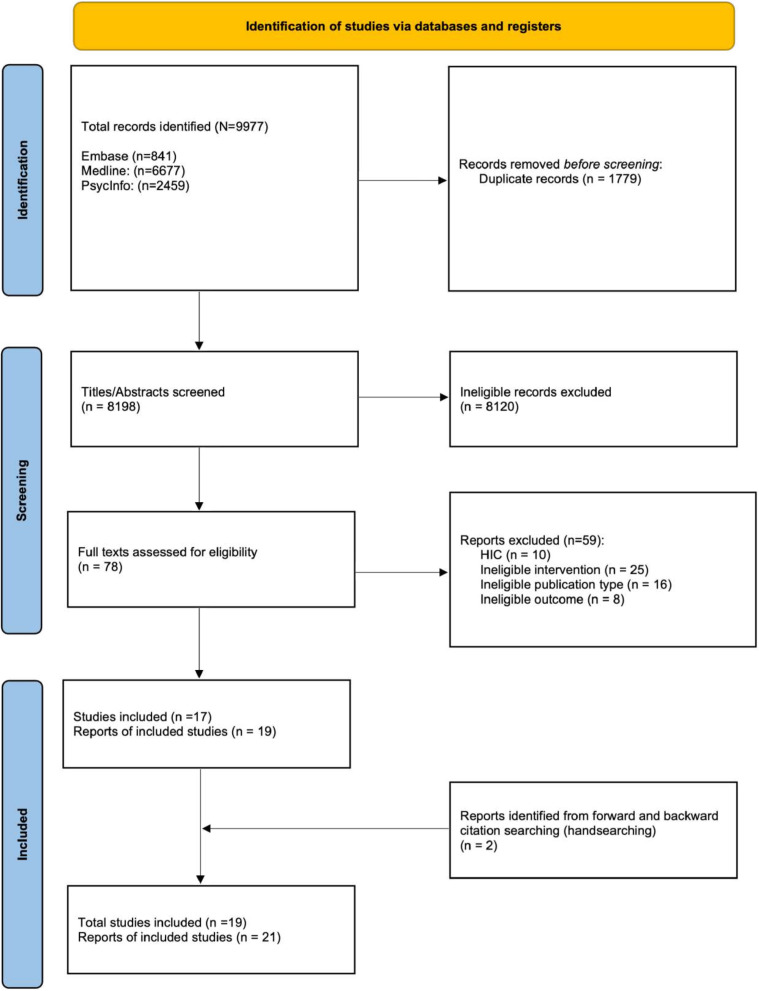
Prisma flow diagram

### Risk of bias

Most studies (*n* = 11) had a moderate risk of bias [32–42], out of which eight were RCTs and one each was mixed-methods, quasi-experimental and qualitative. All studies with a high risk of bias (*n* = 4) [43–46] were RCTs. Finally, the six studies with a low risk of bias comprised three RCTs [47–49] and three quasi-experimental studies [50–52]. Each study’s score and appraisal are included in Table 1.

**Table 1:** Risk of bias assessment

Reference	Study design	Percentage (score)	Risk of bias assessment
Andrade *et al*. (2016) [52]	Quasi-experimental	33.33%	Low
Baldin *et al*. (2018) [35]	RCT	69.23%	Moderate
Bedendo *et al*. (2019) [48]	RCT	84.61%	Low
Bedendo *et al*. (2020) [49]	RCT	92.30%	Low
Christoff (2015) [46]	RCT	46.15%	High
Garg *et al*. (2022) [41]	Quasi-experimental	66.66%	Moderate
Ghosh *et al*. (2022) [40]	Mixed-methods	57.14%	Moderate
Go *et al*. (2020) [33]	RCT	69.23%	Moderate
Hahn *et al*. (2023) [34]	RCT	53.84%	Moderate
Harder *et al*. (2020) [43]	RCT	38.46%	High
Ismayilova *et al*. (2018) [44]	RCT	38.46%	High
Kane *et al*. (2022) [32]	RCT	61.53%	Moderate
Louwagie *et al*. (2022) [39]	RCT	69.23%	Moderate
Nadkarni *et al*. (2022) [47]	RCT	84.61%	Low
Nattala *et al*. (2018) [51]	Quasi-experimental	77.77%	Low
Sanchez *et al*. (2018) [38]	RCT	61.53%	Moderate
Schaub *et al*. (2021) [37]	RCT	53.84%	Moderate
Sharma *et al*. (2023) [50]	Quasi-experimental	77.77%	Low
Signor *et al*. (2013) [36]	RCT	61.53%	Moderate
Staton *et al*. (2022) [45]	RCT	30.76%	High
Suryavanshi *et al*. (2022) [42]	Qualitative	50%	Moderate

### Study characteristics

Table 2 summarizes study characteristics and effectiveness outcomes for all experimental and quasi-experimental studies. All, except one (qualitative), studies were either experimental or quasi-experimental in design [42]. Out of all the RCTs (*n* = 15), three were pilot [32, 44, 47] and three were pragmatic [45, 48, 49] trials. Additionally, the sole mixed-methods study [40] and two [50–52] out of four [41, 51] quasi-experimental studies were pilot studies. The ‘Pesquisa Universitária sobre Bebidas’ (PUB) (Undergraduate Alcohol Research) website intervention was evaluated in two reports, first in its entirety [48] and then component-wise [49]. Moreover, two reports used data from the ‘Balada com Ciência’ Portal Survey Study in Brazil [35, 38]. The WHO Alcohol e-Health portal intervention was evaluated in one pilot study from Brazil [52] and a multi-country RCT [37]. Nine studies each belonged to upper-middle [35, 36, 38, 39, 44, 46, 48, 49, 52] and lower-middle [33, 40–43, 45, 47, 50, 51] countrie,s, and one study included countries from both settings [37]. Only two studies were set in low-income countries [32, 34].

**Table 2:** Study characteristics and effectiveness of experimental and quasi-experimental trials

First author (year) Country	Setting, Sample	Target condition	Study design	DI & platform	Control (or non-digital comparators)	Sample size	Mean (SD) age in years Gender	Effectiveness
Andrade *et al*. (2016) [52] Brazil (Upper-middle)	People who accessed the website and met the cut-off AUDIT score (no other eligibility criteria or recruitment details were provided)	Dependent, harmful alcohol use & heavy, hazardous drinking	Pilot study (quasi-experimental)	Web-based self-help portal named ‘Bebermenos’ meaning ‘Drink Less’ delivered via computer	N/A	929 Low-risk users (LRU) = 319 HHU = 298 SDU = 312	LRU: 40 (11) 56% female (F) HHU: 38 (10) 47.3% F SDU: 40 (11) 35.8% F	No significant differences were detected between HHU and SDU groups following the end of the intervention (*P* = 0.26). Both groups showed significant reduction in alcohol consumption compared to baseline levels (*P* = 0.02). HHU group reduced consumption by 44% and SDU reduced it by 58% and these reductions were maintained at the 1-month follow-up.
Baldin *et al*. (2018) [35] Brazil (Upper-middle)	Nightclubs (adults recruited from queues to enter parties)	BD	RCT (individual)	Web-based intervention to reduce BD: PNF (digital platform unspecified)	Control: only questionnaire (no intervention)	465 Intervention = 224 Control = 241	24.7 (6.0) 35.5% F	No significant between-arm differences observed.
Bedendo *et al*. (2019) [48] Brazil (Upper-middle)	Colleges (18–30-year-old students)	Alcohol use	RCT (individual; pragmatic)	Assessment of motivation + PNF using PUB website (digital platform unspecified)	Control: assessment of motivation only	4460 Intervention = 1725 (Motivated = 1360; Low-motivated = 365) Control = 2735 (Motivated = 2192; Low-motivated = 543)	Motivated = 21.8 (3.0) 52.9% F Low-motivated = 22.2 (3.1) 50.6% F	DI group showed significant reduction in typical number of drinks at 1 month [odds ratio (OR) = 0.71 (0.57–0.88) *P* = 0.002], 3 months [OR = 0.60 (0.45–0.80) *P* < 0.001], and 6 months [OR = 0.68 (0.50–0.93) *P* = 0.016] compared to the control group. However, they also showed a significant increase in the number of alcohol-related consequences at 3 months (*P* = 0.05) compared to the control group. Results were robust up to 3 months and DI was not significantly better than control at 6 months after doing attrition analysis.
Bedendo *et al*. (2020) [49] Brazil (Upper-middle)	Colleges (18–30 year-old students)	Alcohol use	RCT (individual; pragmatic)	PUB website (same as above), which automatically randomized participants into: Arm 1: Full PNF Arm 2: NFO Arm 3: CFO (digital platform unspecified)	N/A (all three arms were digital)	5476 Arm 1 = 1725 Arm 2 = 1800 Arm 3 = 1951	21.8 (3.0) 51.6% F	Individual components were found more effective than the full PNF at multiple follow-up intervals. There were significant effects on AUDIT score by NFO at 1 month [*b* = −0.23, 95% confidence interval (CI) = −0.46; −0.002] (*P* = 0.048) and CFO at 3 months (*b* = −0.33, 95% CI = −0.62; −0.03) (*P* = 0.03). Overall both NFO and CFO reported lower AUDIT scores compared to full PNF throughout the study period. Moreover, NFO reduced number of consequences at 1 month (*b* = −0.16, 95% CI = −0.25; −0.06) (*P* = 0.001) and drinking frequency at 3 months [adjusted OR (aOR) = 0.66 (0.45–0.95) *P* = 0.03], and CFO reduced drinking frequency at 3 months [aOR = 0.69 (0.48–0.99); *P* = 0.045] compared to the full PNF. However, the number of typical drinks was found to be higher in NFO compared to full PNF at 6 months [aOR = 1.46 (1.04–2.05) *P* = 0.03].
Christoff (2015) [46] Brazil (Upper-middle)	Two public and private universities (undergraduate students, ≥18 years of age)	Moderate or high-risk alcohol use	RCT (individual)	Arm 1: computer-based intervention programme, called ASSIST /Motivational BI (ASSIST/MBIc)	Arm 2: feedback plus MBI in an interview (ASSIST/MBIi) Arm 3 (control): receiving only feedback about their ASSIST scores	333 Arm 1 = 128 Arm 2 = 106 Arm 3 = 99	24 (5.4) 57.7% F	No significant between-arm differences observed.
Garg *et al*. (2022) [41] India (Lower-middle)	Outpatient department of one community health centre and two primary health centres (PHCs) + a general hospital and a rural community (adults recruited from all settings)	Alcohol use disorder	Before-after uncontrolled treatment cohort (quasi-experimental)	Tele-psychiatry delivered through various online platforms (web application called 3 AM therapy, Skype and WhatsApp) and phone voice calls in case of no internet	N/A	161 (total) Seven (with AUD)	49.6 (14.5) 63% F	Mean (SD) AUDIT scores reduced drastically and significantly (*P* = 0.001) between the baseline [21.50 (2.35)] and 3-month follow-up [9.83 (4.17)]. However, only six people provided follow-up data and the sample size was too small to draw meaningful conclusions.
Ghosh *et al*. (2022) [40] India (Lower-middle)	Five co-educational colleges in a city (18–21 year-old students)	Harmful and hazardous alcohol use	Mixed-methods (explanatory) study: RCT (cluster; pilot) + Qualitative explanatory study	Web portal or android application-delivered PNF-based BI + digital screening (delivered via any device with internet access)	Control: Screening + brief education about alcohol	25 Intervention = 12 Control = 13	19.6 (2.6) 46% F	No significant between-arm differences observed.
Go *et al*. (2020) [33] Vietnam (Lower-middle)	An antiretroviral therapy (ART) study clinic (enrolled adults receiving ART)	Hazardous alcohol use	RCT (individual)	Arm 1 (BI): Elements of CBT and motivational enhancement therapy (MET) delivered in 2 individual face-to-face sessions and 2 individual booster telephone sessions + standard Ministry of Health (MoH) recommendation	Arm 2 (combined group): CBT + MET in 6 individual face-to-face sessions delivered 1 week apart and 3 optional group sessions delivered concurrently + standard MoH recommendation Arm 3: Standard of care (SOC) + standard MoH recommendation	440 Arm 1 = 147 Arm 2 = 147 Arm 3 = 146	40.2 (5.8) 3.2% F	Both DI and combined groups increased days of abstinence and reduced alcohol consumption compared to SOC. Mean [standard error (SE)] number of drinks per drinking day in DI [3.4(0.3)] and combined groups [2.9 (0.2)] was relatively lower than SOC (4.2(0.3)]. The mean number of heavy drinking days (NHDs) was also lower in the DI and combined groups [3.7(0.7) and 3.4(0.7) days, respectively] compared to SOC [6.7(1.0) days].
Hahn *et al*. (2023) [34] Uganda (Low)	HIV clinic at a hospital (adults with HIV and AUD; prescribed ART for at least 6 months)	Unhealthy alcohol use i.e. the entire spectrum of alcohol use that is harmful to health	RCT (individual)	Arm 1 (Live-call arm): in-person brief workbook-based alcohol counselling + interim boosters delivered by phone Arm 2 (Technology arm): in-person brief workbook-based alcohol counselling + boosters delivered via two-way automated calls either by SMSs or IVR (cell phone)	Arm 3 (control): Standard of care (including brief advice) + wait-listed intervention	269 Arm 1 = 90 Arm 2 = 90 Arm 3 = 89	40.2 34.6% F	Significant differences (*P* < 0.001) in mean number of days drinking (NDDs) compared to control were reported in the live-call arm (3.5) and technology arm (3.6). Mean AUDIT-consumption (AUDIT-C) scores and NDDs were also significantly lower in the live-call [AUDIT-C: 2.3 (*P* < 0.001); NDDs: 4.4 (*P* = 0.002) and technology [AUDIT-C: 2.2 (*P* < 0.001); NDDs: 4.3 (*P* = 0.003)] arms compared to control arm. There was also a 28.9 and 24.9% significant (*P* < 0.001) reduction in unhealthy alcohol use live-call and technology arms, respectively compared to the control arm.
Harder *et al*. (2020) [43] Kenya (Lower-middle)	PHC (tier 2 facility) (adults)	Alcohol use problems	RCT (individual)	Arm 1: mobile phone call delivered MI	Arm 2 (control): Waitlist (1 month) Arm 3: standard in-person MI	300 Arm 1 = 104 Arm 2 = 104 Arm 3 = 92	38 22% F	At 1 month, mean AUDIT-C score was higher in the waitlist control compared to DI arm [adjusted mean difference (CI): 2.88 (2.11–3.66) *P* < 0.005 (intention-to-treat); 3.60 (2.82–4.38) (follow-up)]. There was no difference between scores of mobile vs in-person MI. At 6 months, only mobile and in-person MI were compared and the results were inconclusive.
Ismayilova *et al*. (2018) [44] Kazakhstan (Upper-middle)	Public schools (adolescents at risk for injection drug use and HIV) (14–17 year olds) and their caregivers	Alcohol use	RCT (individual; pilot)	KFT intervention: computer-based pilot interactive multimedia sessions + usual care	Control: Usual care	181 Intervention = 91 Control = 90	15.27 (1.01) 38.67% F	At the 6 month follow-up, there was a significant reduction in BD in DI-group adolescents (OR = 0.18; *P* = 0.023) compared to control.
Kane *et al*. (2022) [32] Zambia (Low)	Two large, urban public-sector Level 1 facilities (adults with HIV reporting unhealthy alcohol use during a regular HIV care visit)	Unhealthy alcohol use	RCT (individual; pilot)	Common elements treatment approach (CETA) (multisession transdiagnostic CBT approach): First CETA session delivered by phone + BI (single session)	Control: BI alone	160 Intervention = 82 Control = 78	40.2 (9.3) 44% F	At 6 months, the mean reduction in AUDIT score was significantly (*P* < 0.001) greater in the DI compared to the control group [− 3.2 points (− 6.2 to −0.1)] with an effect size of *d* = 0.48.
Louwagie *et al*. (2022) [39] South Africa (Upper-middle)	27 primary care clinics in 3 districts (adult patients who had drug-sensitive pulmonary tuberculosis (PTB) and were on treatment)	Hazardous/harmful drinking but not alcohol dependence	RCT (individual)	ProLife intervention: Brief in-person MI + follow-up SMS text messages (via mobile phone) + usual care	Control: usual care + routine treatment and support offered to patients with TB in South Africa (including HIV testing and counselling, health education, dietetic input, social support, point of care biochemical testing)	574 Intervention = 283 Control = 291	22.5% F Intervention: 38.56 (11.15) Control: 39.37 (12.60)	No significant between-arm differences observed.
Nadkarni *et al*. (2022) [47] India (Lower-middle)	Educational institutions (both males and females) and workplaces & primary health centres (males) (18–65 year olds)	Hazardous drinking	RCT (individual; pilot)	Arm 1: mobile phone-based BI–SMS or IVR calls	Arm 2: face-to-face BI: Based on the WHO Mental Health Gap Action Programme (mhGAP) intervention Arm 3 (active control): BI leaflet	74 Arm 1 = 25 Arm 2 = 24 Arm 3 = 25	32.3 (12.5); 8.1% F	No significant between-arm differences observed.
Nattala *et al*. (2018) [51] India (Lower-middle)	Centre for Addiction Medicine at tertiary care hospital (20–60 year-old patients who were recommended inpatient management by the psychiatrist)	Alcohol dependence	Prospective study (quasi-experimental)	Video-enabled cue-exposure-based intervention (VE-CEI) showing video clips containing strategies to deal with various alcohol cues. Videos were displayed using a laptop and Liquid Crystal Display (LCD) projector + treatment as usual (TAU)	TAU: Intensive inpatient programme, including psychosocial interventions about harmful effects of alcohol use, advice about quitting and managing drinking, behavioural rehearsals, medication and detoxification. Contact was maintained via telephone and text messages post-discharge	85 VE-CEI = 43 TAU = 42	37.87 (7.98) 0% F	At 6 months, the DI reported a significantly greater reduction (*P* < 0.0001) than TAU in the mean (SD) quantity of alcohol consumed [4529.0 (2711.0)] and change in NDDs [18.0 (12.0)]. A higher change in mean (SD) quantity of alcohol consumption per day between baseline and follow-up was reported for the DI [25.73 (35.70)] compared to TAU [107.45 (76.10)] (*P* < 0.0001). Mean (SD) time taken (in days) to consume the first drink was significantly (*P* = 0.002) higher in the DI [50.69 (48.53)] compared to TAU [19.72 (22.01)]. DI group also took significantly (*P* < 0.0001) longer (in mean days) [122.11 (27.24)] than TAU [36.56 (28.73)] to consume 50% or more alcohol per day compared to baseline quantity.
Sanchez *et al*. (2018) [38] Brazil (Upper-middle)	Nightclubs (adults recruited from queues to enter parties)	Alcohol consumption	RCT (individual)	Web-based intervention to prevent alcohol abuse: PNF (digital platform unspecified)	Control: Screening only	1057 Intervention = 515 Control = 542	42.5% F	No significant between-arm differences observed.
Schaub *et al*. (2021) [37] Belarus (Upper-middle) Brazil (Upper-middle) Mexico (Upper-middle) India (Lower-middle)	Community samples recruited via information flyers and newspapers, magazines, radio, social media, websites and informational events related to alcohol and health (18–75 year olds)	Drinking patterns considered harmful, hazardous or suggestive of dependence	RCT (individual)	Web-based Alcohol e-Health program: an accessible interactive self-help tool for people seeking to reduce or discontinue their use of alcohol (digital platform unspecified)	Waiting list (control): Participants were told they would be provided access to the program in 6 months and were referred to a web page with information about risk factors of alcohol dependence and its effect on the brain and body, types of alcoholic beverages, standard drink definitions, social effects of drinking and alcohol use in women and adolescents.	1400 Brazil = 587 [intervention group (IG) = 290; control group (CG) = 297] Mexico = 509 [IG = 256; CG = 253] India = 212 [IG = 95; CG = 117] Belarus = 92 [IG = 46; CG = 46]	37.6 (10.5) 29.9% F	At 6 months, a significant decrease (*P* < 0.001) in mean (SD) AUDIT score (complete-case analysis) was observed in the DI group [7.4 (7.8)] compared to the control group [3.2 (7.1)]. There was also a significantly higher reduction (*P* = 0.005) in mean (SD) standard drinks consumed in the DI [24.7 (39.6)] over the control group [15.4 (29.4)]. Moreover, 25.6% (intervention) versus 12.7% (control) participants had a total AUDIT score below 8 (not harmful/hazardous) after 6 months.
Sharma *et al*. (2023) [50] India (Lower-middle)	Centre for Addiction Medicine at tertiary care hospital (adults seeking treatment)	Alcohol dependence syndrome (ADS)	Pilot implementation (quasi-experimental)	Smartphone-based mobile application ‘Quest’ for relapse prevention (aftercare) in patients diagnosed with alcohol dependence + TAU	Control: TAU	30 Intervention = 15 Control = 15	Intervention = 33.27 (6.20) Control = 31.80 (4.91) 0% F	No significant between-arm differences observed.
Signor *et al*. (2013) [36] Brazil (Upper-middle)	Population seeking help from a telephone information service for alcohol use cessation	Alcohol misuse	RCT (individual)	Telephone-delivered brief MI + self-help material	Control: TAU + self-help material + interviews using a closed-ended questionnaire + written advice only	637 Intervention = 293 Control = 344	29% F	Significant differences were observed in alcohol consumption abstinence between the DI and control groups at 6 months [OR = 1.7 (1.0–2.7); *P* = 0.01]. 70% of participants in the DI group reported alcohol abstinence compared to 41% in the control group. The control group was more likely to relapse than the intervention group [OR = 2.5 (1.1–5.3); (*P* = 0.01)].
Staton *et al*. (2022) [45] Tanzania (Lower-middle)	Major medical referral centre [adult patients seeking care at a for an acute (<24 hours) injury]	Harmful and hazardous drinking	RCT (individual; pragmatic)	Mobile-phone-based SMS-integration (booster) into a nurse-led brief negotiation intervention [‘Punguza Pombe Kwa Afya Yako’ (PPKAY)/‘Reduce Alcohol for Your Health’ intervention (PPKAY)] Arm 1: PPKAY with standard booster Arm 2: PPKAY with personal booster	Arm 3: PPKAY without booster	41 Arm 1 = 23 Arm 2 = 18	Arm 1: 37 (15) 4% F Arm 2: 31 (12) 0% F	Only feasibility outcomes measured.


The qualitative study (based in India) targeted ‘unhealthy alcohol use’ [42] in people with HIV and recruited adults from a government tertiary care hospital. It used in-depth interviews (IDIs) and focus-group discussions (FGDs) to assess the acceptability, adaptability and feasibility of a computer-based BI delivered by a virtual counsellor (VC) through a software platform. The IDIs had 10 participants (10% female) and FGDs consisted of healthcare providers (three females, six males), research counsellors (12 females, one male) and alcoholics anonymous group members (*n* = 12 males).

### Intervention content

Table 3 includes details of intervention content and related outcomes. Web-based personalized normative feedback (PNF) interventions broadly consisted of four components across studies. First, personalized feedback on participants’ alcohol consumption levels based on Alcohol Use Disorders Identification Test (AUDIT) scores, drinking frequency, etc. Second, normative comparisons i.e. examining the participants’ consumption levels against other people of similar demographics. Third, personalized estimates of expenditure on alcohol consumption, and fourth, information on consequences of drinking and guidance to cope with relapse or reduce consumption. Interventions that solely provided personalized feedback were more interactive and involved goal-setting, documentation and automated personalized feedback to achieve goals. One of the interventions [40] also incorporated goal-setting along with personalized and normative feedback.

**Table 3:** Intervention characteristics and implementation/intervention outcomes

**First author (Year)**	DI content	Delivery agent (if applicable)	Duration	Intervention/implementation outcomes
Andrade *et al*. (2016) [52]	The self-help web-portal operated in three stages: ‘Preparing for action’, ‘Goal-setting’ and ‘Action.’ Participants analyse past alcohol use, set goals, self-monitor, discuss, do cognitive exercises and keep an updated diary. Automated feedback was provided to reduce risky drinking and achieve goals.	–	6 weeks	Intervention was acceptable but had low adherence (76.9% dropout rate).
Baldin *et al*. (2018) [35]	Customized normative feedback screen consisted of four parts: (i) feedback on alcohol consumption level at assessment and information on complications (mental, physical, etc.) according to each risk level (ii) social norms information using general population data to highlight the atypical drinking behaviour of the participant (iii) personalised estimates of financial burden pertaining to alcohol per month and year (iv) general information backed by data to reduce consequences of alcohol consumption.	–	Applied after initial evaluation	–
Bedendo *et al*. (2019) [48]	The website was based on PNF and its main components were assessment and feedback. It established a drinking profile (based on drinking frequency, AUDIT scores, etc.), made gender-specific normative comparisons and provided information on practical and health costs of alcohol, possible negative consequences and strategies to avoid risky drinking.	–	5–10 minutes to complete the full intervention (based on pilot data)	–
Bedendo *et al*. (2020) [49]	Components-evaluation of the website above. PNF included all the components described above. NFO include only drinking profile, gender-specific comparisons and strategies to avoid risky drinking. CFO comprised of practical and health costs, alcohol-related and socio-environmental consequences and strategies.	–	Unspecified	–
Christoff (2015) [46]	The computer-based MI was a simple interactive website consisting of the intervention content across different web pages. The main elements of the intervention were Feedback, Responsibility, Advice, Menu of Options, Empathy and Self-Efficacy (FRAMES). It aimed to promote self-management skills and behaviour change by raising awareness about risks of substance use. It was designed to be linked to the ASSIST, allow participants to report problems, provide advice, coping skills and education about the substance(s) involved.	–	20 minutes (approximately)	–
Garg *et al*. (2022) [41]	Tele-psychiatry facilitated through an online platform with features including appointment-scheduling and tracking, video-conferencing, session timer, history and note-taking.	Two psychiatrists + 4 lay counsellors	20–30 minutes	Participants on average were satisfied with the sessions and the platform. They felt comfortable and were willing to use telemedicine in the future.
Ghosh *et al*. (2022) [40]	Web portal or android application provided personalised and normative feedback. Personalised feedback was based on AUDIT scores, history of alcohol use and mental disorders. Normative feedback was about the risks of alcohol use using pictorial representations of harm. Participants could choose goals from options and could watch a video on drink-refusal skills.	–	10–15 minutes	Most participants ‘strongly agreed’ that the intervention was appropriate. Acceptability/feasibility were measured through total logins and total completed screenings (96.5%). Everyone completed the intervention.
Go *et al*. (2020) [33]	Booster (phone) sessions were delivered. The general BI sessions included CBT, skill-building to refuse alcohol, manage cravings and develop self-efficacy. They also included information about alcohol effects and behaviour change strategies. Specific phone-based content was not outlined.	Paraprofessional counsellors	Two sessions occurring 2–3 weeks after each in-person session	Telephone-session outcomes were not reported separately.
Hahn *et al*. (2023) [34]	The non-DI included a workbook that participants used to set a maximum drinking goal for themselves. The telephone or SMS delivered booster sessions were tailored to participants’ goals and aimed to check progress, provide positive reinforcement and encouragement. They also allowed for a revision of goals.	Lay health counsellor	Live call-arm: every 3 weeks Technology arm: Twice-weekly	86.5 and 44.7% of scheduled booster calls were completed in live-call and technology arms, respectively.
Harder *et al*. (2020) [43]	MI (mobile-delivered) included affirmations, reflective listening, open-ended questions and summarization to motivate participants to change alcohol use patterns.	Trained clinicians	Single session 30 minutes	–
Ismayilova *et al*. (2018) [44]	KFT intervention included three multimedia sessions on risk reduction, self-efficacy, resistance to peer pressure and parent–child communication and support. It used a simulated human-interaction platform wherein participants confronted several risky situations including substance use related scenarios. Each scenario allowed the user to communicate with a youth or caregiver avatar to practise interpersonal skills like empathy, assertiveness, etc. Each caregiver–youth pair shared a computer to engage with the intervention’s exercises and discussions.	–	Weekly sessions (25–30 minutes)	Participants found the intervention’s computerized platform to be engaging, self-pacing and confidential. They also found the content relatable.
Kane *et al*. (2022) [32]	CETA (phone-based) consisted of nine CBT elements: engagement, introduction/psychoeducation, safety, substance use reduction, cognitive coping and restructuring, problem solving, behavioural activation, relaxation and exposure (live and imaginal).	Lay counsellors	1 hour weekly sessions for 6–12 sessions (dependent on clinical complexity and response)	–
Louwagie *et al*. (2022) [39]	SMS messages were sent as follow-up reinforcements for the in-person MI component. Seven alcohol-related messages containing information, motivation and behavioural skills were sent.	–	Two times per week over 12 weeks	Fidelity was measured in terms of SMS delivery. 80.4% of messages were delivered to 41.9% participants who completed the first MI.
Nadkarni *et al*. (2022) [47]	BI included text messages and IVRs on self-awareness, motivation, self-reflection, safe drinking, drinking and risk management, drinking alternatives, health education, personalised feedback and goal setting. Messages were either in English or a vernacular language of the participants’ choice. Most messages did not require a response.	–	2–3 times a week over 8 weeks	The delivery of a mobile-delivered intervention was found feasible in people with hazardous drinking and potentially acceptable as well.
Nattala *et al*. (2018) [51]	VE-CEI consisted of live-action videos in regional languages involving local theatre artists shot in simulated settings mimicking real-life. Scenes were based on patients’ experiences and each video dealt with managing triggers around one of the following alcohol cues: craving, social pressure, poor coping with negative situations, physical fatigue, boredom, stress, anger and alcohol lapse. The 8th video depicted management techniques in case drinking occurs.	Implemented by a registered psychiatric nurse (first author)	Eight group sessions over 3 weeks (45 minutes–1 hour per session)	–
Sanchez *et al*. (2018) [38]	Web-based PNF screen consisted of: (i) AUDIT score explanation along with the associated health risk (ii) breathalyzer information along with accident and sex-related risks (iii) monetary expenditure related to alcohol (monthly and yearly) (iv) comparison of consumption with other people of similar demographics (v) web-page with information was provided with resources to inform decision-making about alcohol consumption	–	Applied at baseline	–
Schaub *et al*. (2021) [37]	Web-based, interactive self-help tool (Alcohol e-Health program) with a comprehensive diary as its core element. Participants filled it out daily by dragging and dropping icons of country-specific drinks and documenting each consumption occasion in terms of when, where, what and how much they drank and with whom. They could also set goals and document their feelings about drinking. Participants could explore advantages and disadvantages of drinking and find guidance to cope with relapse and resist social pressure. Diary data was used to automatically generate personalised weekly feedback regarding drinking goals.	–	6 weeks	Higher satisfaction [mean (SD) CSQ-8* scores] with study participation was reported in the intervention group [21.56 (4.11)] compared to controls [18.92 (4.65)] (*P* < 0.001). 37.6% participants had at least one diary entry and 6% completed the relapse tool. 71.9% participants did not report any adverse effects during the study.
Sharma *et al*. (2023) [50]	‘Quest’ application content was divided into two modules: (i) ‘Learn’ (knowledge repository) consisting of educational materials (supplemented with videos and pictures) on drinking, effects on the body and treatments available (ii) ‘My Quest’ containing relapse-prevention components divided into five submodules: daily diary, reflect & plan, setting goals, schedule and activities.	–	Engage with application once daily for best results	High usability [mean (SD) 5.80 (0.42) out of 7] and acceptability (by 65% users) was reported. Engagement decreased over time.
Signor *et al*. (2013) [36]	Telephone-based MI (content specifications not provided).	Counsellor (university students drawn from health programmes)	30 minutes (approximately)	–
Staton *et al*. (2022) [45]	SMS-boosters reinforcing knowledge of alcohol-related harms, and the self-efficacy and goal-setting components of the PPKAY intervention through short, personalised phrases.	–	12 messages over a 3 month period	SMS-booster delivery was found to be feasible and highly acceptable for recipients. Intervention fidelity (delivery of boosters) was moderate.
Suryavanshi *et al*. (2022) [42]	Computer-based BI was delivered by the VC using multiple-choice questions and CBT techniques including personalised feedback, problem-solving for high-risk situations, goal-setting for alcohol use and information-provision about drinking-consequences.	VC: 3D parrot (named Peedy) with human-like mannerisms and speech (coded through text-to-speech software). It has 50+ actions that can be chosen to convey relevant emotions	Single session (20 minutes approximately)	The intervention was acceptable, appropriate and feasible. Most participants preferred a combination of virtual and human counselling, and some adaptations were suggested for improvement of the intervention.

^*^CSQ-8**:** Client Satisfaction Questionnaire-8

Content specifications of digitally-delivered motivational interview (MI) were provided only in two studies [43, 46]. They both shared features of reflection, awareness and empathy to promote behaviour change. Apart from MI, studies used Bis, which included digitally-delivered cognitive behavioural therapy (CBT) [32, 33, 42] and short message service (SMS)-based motivation and feedback [34, 39, 45, 47]. CBT across studies shared features of psychoeducation, problem-solving, promotion of self-efficacy and cognitive restructuring. SMS-based motivation included affirmations and reinforcement of alcohol-related goals and harms.

### Effectiveness

Interventions with positive outcomes most commonly pertained to a reduction in AUDIT scores, number of drinking days and unhealthy alcohol consumption, along with an increase in abstinence. WHO’s Alcohol e-Health programme was found effective in both its Brazilian pilot (significant [*P* = 0.02] reduction in alcohol consumption compared to baseline: 44% [hazardous users (HHU)] and 58% [Suggestive of dependence users (SDU)]) [52] and updated multi-country version (mean(SD) decrease in AUDIT score for DI group [7.4 (7.8)] compared to the control group [3.2 (7.1)]) [37]. However, other versions of the web-based PNF either did not significantly improve alcohol outcomes [35, 38, 40] or were only partially effective [48]. For instance, the PUB was initially effective in reducing alcohol consumption (odds ratio (OR) = 0.60 [0.45–0.80] *P* < 0.001), but the effect was attenuated due to attrition at follow-up [48]. Notably, a component evaluation of PUB revealed that individual components like normative feedback only (NFO) and consequences feedback only (CFO) in isolation were more effective than the full PNF in decreasing AUDIT scores (‘NFO’ at 1 month [*b* = −0.23, 95% confidence interval (CI) = −0.46; −0.002] [*P* = 0.048]; ‘CFO’ at 3 months [*b* = 0.33, 95% CI = −0.62; −0.03] [*P* = 0.03]) and drinking frequency at 3 months (‘NFO’: adjusted OR (aOR) = 0.66 [0.45–0.95] *P* = 0.03; ‘CFO’: aOR = 0.69 [0.48–0.99]; *P* = 0.045) [49].

MI was assessed in three studies, and one of them found significant differences between intervention and control groups (OR = 1.7 [1.0–2.7]; *P* = 0.01) in alcohol consumption, abstinence and relapse in favour of the intervention [36]. However, results from the other studies were mixed. A phone-based MI intervention in Kenya was found superior to waitlist controls (adjusted mean difference (CI): 2.88 [2.11–3.66] *P* < 0.005 (intention-to-treat); 3.60 [2.82–4.38] (follow-up)) but showed no difference when compared to in-person MI [43]. A broader study with the aim of reducing several types of substance use found that while MI reduced alcohol-specific scores, there was no significant overall between-group difference in Alcohol, Smoking and Substance Involvement Screening Test (ASSIST) scores [46].

Three studies used Bis, including digitally-delivered CBT, skill-building and behaviour change [32, 33, 42]. Even though only two tested for effectiveness, the results were overwhelmingly positive in improving several outcomes, including AUDIT scores (−3.2 points [−6.2 to −0.1]) [32], alcohol consumption, number of drinking and heavy drinking days and abstinence [32, 33]. Additionally, an innovative multimedia-based behaviour-change intervention based on developing self-efficacy and fostering family support significantly reduced BD in adolescents compared to usual care (OR = 0.18; *P* = 0.023) [44]. However, it must be noted that this was a pilot study, and these results were based on a small sample (*N* = 181).

SMS-based motivation and feedback were used in four studies [34, 39, 45, 47] but its effectiveness was assessed in three. The only study that found significant proof of effectiveness used SMS and phone-call-based boosters to supplement a larger intervention [34]. It found that mean AUDIT-C scores and number of drinking days (NDDs) were significantly lower in the live-call [AUDIT-C: 2.3 (*P* < 0.001); NDDs: 4.4 (*P* = 0.002) and technology [AUDIT-C: 2.2 (*P* < 0.001); NDDs: 4.3 (*P* = 0.003)] arms compared to control arm [34]. Both Nadkarni *et al*. [47] and Louwagie *et al*. [39] did not find significant differences in intervention and control groups. The former delivered a BI using SMS and interactive voice response (IVR) calls, but it was a pilot RCT and, therefore, was not powered to detect effectiveness. The latter used SMS follow-ups as reinforcement for in-person MI, which implies that the results are not solely reliant on the digital component.

In India, an online telepsychiatry platform drastically reduced the mean AUDIT scores (baseline = 21.50 [2.35] versus 3-month follow-up = 9.83 [4.17]). However, only six people’s follow-up data was provided and this sample size was too small to draw conclusions [41]. Additionally, a mobile application based on knowledge dissemination and relapse prevention was not significantly different compared to usual treatment [50], but the latter was a pilot study, and it found a decrease in number of drinking days within both intervention and control groups in three months, compared to baseline (values not reported in the study). Finally, a cue-exposure intervention involving videos based on participants’ real lives was found effective in reducing the mean quantity of alcohol consumption per day (DI = 25.73 [35.70]; TAU = 107.45 [76.10]; *P* < 0.0001) and delaying it for longer as compared to usual treatment [51].

### Implementation outcomes

The main implementation outcomes were feasibility [40, 42, 45, 47], acceptability [40, 42, 45, 47, 50, 52], engagement [44, 50], adherence [34, 37, 52], fidelity [52, 58], satisfaction [37, 41], appropriateness [40–42], adverse effects [37] and usability [50]. Their measurement and conceptualisation varied across studies. Overall, interventions reported high acceptability, feasibility, satisfaction and appropriateness. Fidelity was measured in terms of SMS delivery in both Staton et al. [45] and Louwagie *et al*. [39], with moderate to low results. Engagement was tested for the Kazakhstani Family Together (KFT) intervention [44] and ‘Quest’ application [50] using focus-group interviews and application engagement days, respectively. While participants found KFT’s computerized format engaging, engagement with Quest decreased over time. Adherence was measured in terms of percentage of the intervention completed for Hahn *et al*. [34] and Schaub *et al*. [37] (scheduled booster calls and diary entries, respectively) and dropout rate for Andrade *et al*. [52]. Schaub *et al*. [37] and Andrade *et al*. [52] reported low adherence, while Hahn *et al*. [34] had mixed results such that the live-call arm had high adherence and the technology arm had low adherence.

## DISCUSSION

This is the first systematic review to summarize the evidence on DIs for alcohol use and AUDs in LMICs in terms of their characteristics, content, effectiveness and implementation outcomes. Several DIs were identified, ranging from simple digitally-delivered BIs, MI or counselling to more advanced websites, mobile applications and multimedia-based interventions.

Previous research in HICs reported high acceptability of mHealth [53] and technology-assisted interventions [54], but there was insufficient evidence about feasibility and other implementation outcomes. Most interventions in our review reported high acceptability but also provided evidence of high feasibility. Bonfiglio *et al*. [55] and Holmes *et al*. [54] both found low or mixed effectiveness of DIs for substance use in HICs, given the weak quality of studies and lack of adequate comparisons. Similarly, we found mixed results for effectiveness, such that positive and partially positive results outnumbered the negative, but study designs and quality did not allow for robust conclusions to be drawn. A previous review on the effectiveness of computer-delivered interventions (CDIs) for reducing alcohol consumption in HICs [12] found small but significant improvements in alcohol use outcomes but reported larger effects when personal contact was provided along with the CDI. This was observed in our review as well, wherein interventions with a component of human-involvement as part of the DI showed more positive effectiveness outcomes as compared to standalone interventions like websites and mobile applications. In fact, one of the studies delivered a BI using a VC and a majority of participants felt that a combination of human and virtual counselling would have been preferable over virtual counselling alone. Finally, a Cochrane review on personalized DIs in HICs found almost no evidence of a difference in effectiveness between digital and face-to-face interventions for reducing harmful alcohol consumption [15]. While very few reports made that comparison in the present review, no significant differences were found between digital and face-to-face BI [47] and MI (mobile-based) [43], which could imply that DIs could be potential replacements for in-person treatment without compromising on quality. However, this was based on short-term assessments and more evidence is required for establishing the comparative effectiveness of DIs against non-digital treatment.

Our review had several limitations. It did not use the quality appraisal of studies to dictate their eligibility for inclusion in the review or their subsequent analysis. Additionally, scoring of the quality appraisal was not weighted, i.e. the scores were not based on the relative importance of each question in terms of study design. Due to time constraints, double data extraction and hand-searching were not conducted. However, the cross-check method and citation-chaining were employed, respectively. Moreover, the search was limited to three databases and the strategy did not seek non-English studies and grey literature. While our search found that much of the literature on DIs is predominantly in English, our search strategy was not designed to identify academic literature in other languages, such as Portuguese and Spanish, which are prevalent in LMICs. Additionally, the exclusion of grey literature may have led to the omission of relevant unpublished or less accessible studies. In terms of the synthesis, no common rubric was established to compare effectiveness outcomes, the groupings that we prioritized were subjective to some extent, a theory of change for the interventions was not developed, heterogeneity was not explored statistically, and the robustness of the synthesis itself was not assessed. Studies included in the review also had several limitations, like small sample sizes [39, 40, 47, 51, 52], high attrition [36, 38, 48, 49] and reliance on self-report (which increased the risk of social desirability bias). Another limitation of effectiveness studies was the short duration of follow-up periods, wherein most studies had a maximum follow-up of either three or six months. Additionally, there was no standardization of intervention characteristics (For instance, there were three studies assessing the effectiveness of digitally delivered MI, but one of them did not specify the content [36] and the other two differed in their approaches [43, 46]. In turn, even for studies that supposedly implemented the same intervention, comparisons were difficult since even a few differing components could impact the outcomes) and definitions and measurement of outcomes. For instance, the cut-off AUDIT score for harmful drinking differed across studies [35, 48].

The generalisability of our findings could be limited by a few factors. First, the under-representation of women was true in most included studies, such that some had no women at all [45, 50, 51]. This could potentially be attributed to lower consumption rates in some cases, but it could also be a product of under-reporting, stigmatization and barriers to help-seeking [56]. Second, samples recruited from, for instance, queues to enter nightclubs [35, 38] or by offering internships to college students [48, 49] were prone to selection bias. Third, amongst studies with positive results, several were based on individuals either with HIV [32–34] or at risk for it [44], and others varied in terms of the intensity of alcohol use they were assessing. For instance, while some populations were seeking help from a telephone service [36] or a website [52], others had recommended inpatient care [51]. Therefore, the delivery settings and conditions of interest differed significantly, and there is not enough positive evidence for generalization to any particular population except for those affected by HIV. Finally, our findings have limited generalisability in low-income countries since only two studies were identified from there.

Despite these limitations, our review followed PRISMA guidelines, had a clear scope and predefined eligibility criteria, and is reproducible because of its protocol-driven approach. We used several past reviews to construct a comprehensive search strategy, conducted double-screening and double-quality appraisal and performed backwards and forward searching. This enabled the identification of a significant amount of LMIC-specific research that was not included in any previous evidence synthesis efforts on DIs for substance use. Finally, a risk of bias assessment was conducted to account for methodological and reporting bias in studies.

There are several implications of our review. Future research on DIs needs to focus on improving follow-up, investigating the factors contributing to high attrition rates and evaluating outcomes over a longer time period. Given the weak evidence in favour of standalone DIs, further research on their effectiveness is imperative because the development of websites and mobile applications requires considerable resources. Previous research on DIs for substance uses emphasized the need to focus on implementation processes and address contextual factors [55]. However, several studies in the present review did not evaluate implementation outcomes. Out of the ones that did, there were methodological inconsistencies and errors that hindered analysis [37], use of non-standardized measures [36] and very small and non-representative samples [42]. Therefore, implementation outcomes must be prioritized in subsequent research. Additionally, further research is required to determine the association between DI-effectiveness and intrinsic characteristics of participants, such as motivation levels—as assessed by Bedendo *et al*. [48]. Clinically, there has been an emphasis in past research to move away from tertiary care for alcohol use [3, 22]. According to existing research, interventions for AUDs can be incorporated into primary care to promote effective use of scarce healthcare resources, and this can be supplemented with policy efforts to move towards a stepped-care approach in healthcare provision for AUDs [57]. Additionally, some studies in our review mentioned resource limitations and funding constraints. For instance, the features included in a mobile application had to be limited [50], and one of the trials could not be fully powered because funding constraints led to the recruitment getting suspended early [39]. This highlights the need for policy changes to increase investment in research and development of digital health technologies.

These findings only outline some of the challenges to the implementation and sustainability of DIs in LMICs. Other studies have identified additional barriers, primarily technological and infrastructural limitations such as unaffordability of digital tools (e.g. computers, mobile phones), unreliable internet and electricity access and challenges with system usability, including lack of knowledge and inadequate training [58, 59]. Linguistic and cultural factors, such as beliefs and attitudes, perceptions of technology and digital divides amongst various sections of society, can also pose barriers to adoption [59]. Despite these obstacles, the current research reflects a push towards the strategic implementation and scaling up of DIs globally. Some key enablers for effective implementation of eHealth interventions in LMICs (particularly Asia and Africa) include compatibility of the interventions with the needs and priorities of local populations, strong government involvement and local ownership, alignment with national health system strategies, and collaboration across public, private and non-profit sectors [60]. The WHO’s ‘Global Strategy on Digital Health 2020–25’ outlined a detailed action plan to implement digital technologies globally to build sustainable and cost-effective digital health ecosystems that promote digitalization of the health sector and promote healthier populations [61]. This framework can serve as a guide for effective implementation and adoption of DIs in LMICs, ensuring alignment with and consideration of local and global priorities.

Research from LMICs has rarely featured in past evidence synthesis efforts on DIs, and this was the first review to focus solely on alcohol use in LMICs. While the evidence for effectiveness of DIs in LMICs was limited, the database is growing, and increasingly innovative interventions are being developed to address the problem. Given the existing evidence in favour of a human-interactive element, perhaps future interventions should be developed collaboratively in a manner that maximizes benefit while minimizing cost.

## Supplementary Material

Supplementary_Material_Updated_oqaf004

## Data Availability

The data underlying this article are available in the article and in its online supplementary material.
