# Supplementary material for: Digital interventions for alcohol use and alcohol use disorders in low- and-middle-income countries: a systematic review
Source: Oxf Open Digit Health. 2025 Jan 12;3:oqaf004. doi: 10.1093/oodh/oqaf004 (PMC11932145; doi:10.1093/oodh/oqaf004)
Supplement: Supplementary_Material_Updated_oqaf004 [file Supplementary_Material_Updated_oqaf004.docx]

**Digital Interventions for Alcohol Use and Alcohol Use Disorders in Low- and-Middle-Income Countries: A Systematic Review**

Payal Khatore^1^, Hizkia Yolanda^1^, Jaeden Joyner^1^, Abhijit Nadkarni^1,2,3^*

**^1^**Centre for Global Mental Health, London School of Hygiene and Tropical Medicine, Keppel Street, London, WC1E 7HT, United Kingdom

^2^Department of Population Health, London School of Hygiene and Tropical Medicine, Keppel Street, London, WC1E 7HT, United Kingdom

^3^Addictions and Related Research Group, Sangath, Porvorim, Goa, India 403501

*Correspondign author: Department of Population Health, London School of Hygiene and Tropical Medicine, Keppel Street, London, WC1E 7HT, United Kingdom [abhijit.nadkarni@lshtm.ac.uk](mailto:abhijit.nadkarni@lshtm.ac.uk)

**APPENDIX 1: PRISMA Checklist**

| **Section and Topic** | **Item #** | **Checklist item** | **Location where item is reported** |
| --- | --- | --- | --- |
| **TITLE** | | |  |
| Title | 1 | Identify the report as a systematic review. | 1  10 |
| **ABSTRACT** | | |  |
| Abstract | 2 | See the PRISMA 2020 for Abstracts checklist. | 5 |
| **INTRODUCTION** | | |  |
| Rationale | 3 | Describe the rationale for the review in the context of existing knowledge. | 7–9 |
| Objectives | 4 | Provide an explicit statement of the objective(s) or question(s) the review addresses. | 9 |
| **METHODS** | | |  |
| Eligibility criteria | 5 | Specify the inclusion and exclusion criteria for the review and how studies were grouped for the syntheses. | 10–11 |
| Information sources | 6 | Specify all databases, registers, websites, organisations, reference lists and other sources searched or consulted to identify studies. Specify the date when each source was last searched or consulted. | 11 |
| Search strategy | 7 | Present the full search strategies for all databases, registers and websites, including any filters and limits used. | 11–12  39–42 |
| Selection process | 8 | Specify the methods used to decide whether a study met the inclusion criteria of the review, including how many reviewers screened each record and each report retrieved, whether they worked independently, and if applicable, details of automation tools used in the process. | 12 |
| Data collection process | 9 | Specify the methods used to collect data from reports, including how many reviewers collected data from each report, whether they worked independently, any processes for obtaining or confirming data from study investigators, and if applicable, details of automation tools used in the process. | 12 |
| Data items | 10a | List and define all outcomes for which data were sought. Specify whether all results that were compatible with each outcome domain in each study were sought (e.g. for all measures, time points, analyses), and if not, the methods used to decide which results to collect. | 12  43 |
|  | 10b | List and define all other variables for which data were sought (e.g. participant and intervention characteristics, funding sources). Describe any assumptions made about any missing or unclear information. | 12  43 |
| Study risk of bias assessment | 11 | Specify the methods used to assess risk of bias in the included studies, including details of the tool(s) used, how many reviewers assessed each study and whether they worked independently, and if applicable, details of automation tools used in the process. | 13 |
| Effect measures | 12 | Specify for each outcome the effect measure(s) (e.g. risk ratio, mean difference) used in the synthesis or presentation of results. | 12 |
| Synthesis methods | 13a | Describe the processes used to decide which studies were eligible for each synthesis (e.g. tabulating the study intervention characteristics and comparing against the planned groups for each synthesis (item #5)). | 13 |
|  | 13b | Describe any methods required to prepare the data for presentation or synthesis, such as handling of missing summary statistics, or data conversions. | N/A |
|  | 13c | Describe any methods used to tabulate or visually display results of individual studies and syntheses. | 13 |
|  | 13d | Describe any methods used to synthesize results and provide a rationale for the choice(s). If meta-analysis was performed, describe the model(s), method(s) to identify the presence and extent of statistical heterogeneity, and software package(s) used. | 13 |
|  | 13e | Describe any methods used to explore possible causes of heterogeneity among study results (e.g. subgroup analysis, meta-regression). | 13 |
|  | 13f | Describe any sensitivity analyses conducted to assess robustness of the synthesized results. | N/A |
| Reporting bias assessment | 14 | Describe any methods used to assess risk of bias due to missing results in a synthesis (arising from reporting biases). | N/A |
| Certainty assessment | 15 | Describe any methods used to assess certainty (or confidence) in the body of evidence for an outcome. | N/A |
| **RESULTS** | | |  |
| Study selection | 16a | Describe the results of the search and selection process, from the number of records identified in the search to the number of studies included in the review, ideally using a flow diagram. | 14 |
|  | 16b | Cite studies that might appear to meet the inclusion criteria, but which were excluded, and explain why they were excluded. | 14–15  44–47 |
| Study characteristics | 17 | Cite each included study and present its characteristics. | 16–21 |
| Risk of bias in studies | 18 | Present assessments of risk of bias for each included study. | 15–16 |
| Results of individual studies | 19 | For all outcomes, present, for each study: (a) summary statistics for each group (where appropriate) and (b) an effect estimate and its precision (e.g. confidence/credible interval), ideally using structured tables or plots. | 16–21 |
| Results of syntheses | 20a | For each synthesis, briefly summarise the characteristics and risk of bias among contributing studies. | 15–26 |
|  | 20b | Present results of all statistical syntheses conducted. If meta-analysis was done, present for each the summary estimate and its precision (e.g. confidence/credible interval) and measures of statistical heterogeneity. If comparing groups, describe the direction of the effect. | N/A |
|  | 20c | Present results of all investigations of possible causes of heterogeneity among study results. | N/A |
|  | 20d | Present results of all sensitivity analyses conducted to assess the robustness of the synthesized results. | N/A |
| Reporting biases | 21 | Present assessments of risk of bias due to missing results (arising from reporting biases) for each synthesis assessed. | N/A |
| Certainty of evidence | 22 | Present assessments of certainty (or confidence) in the body of evidence for each outcome assessed. | N/A |
| **DISCUSSION** | | |  |
| Discussion | 23a | Provide a general interpretation of the results in the context of other evidence. | 26–27 |
|  | 23b | Discuss any limitations of the evidence included in the review. | 27–28 |
|  | 23c | Discuss any limitations of the review processes used. | 27 |
|  | 23d | Discuss implications of the results for practice, policy, and future research. | 28–29 |
| **OTHER INFORMATION** | | |  |
| Registration and protocol | 24a | Provide registration information for the review, including register name and registration number, or state that the review was not registered. | 10 |
|  | 24b | Indicate where the review protocol can be accessed, or state that a protocol was not prepared. | 10 |
|  | 24c | Describe and explain any amendments to information provided at registration or in the protocol. | 10 |
| Support | 25 | Describe sources of financial or non-financial support for the review, and the role of the funders or sponsors in the review. | N/A |
| Competing interests | 26 | Declare any competing interests of review authors. | N/A |
| Availability of data, code and other materials | 27 | Report which of the following are publicly available and where they can be found: template data collection forms; data extracted from included studies; data used for all analyses; analytic code; any other materials used in the review. | 3 |

**APPENDIX 2: Search Strategy**

|  | **CENTRAL SEARCH STRATEGY (EMBASE)**  **DIGITAL/TECHNOLOGY-BASED INTERVENTION(S)** |
| --- | --- |
| #1 | exp Web Browser/ OR exp Online System/ OR exp Internet/ OR exp Mobile Phone/ OR exp MP3 Player/ OR exp Computer System/ OR exp Mobile Application/ OR exp Microcomputer/ OR exp Reminder System/ OR exp Telemedicine/ OR exp Telecommunication/ OR exp Text Messaging/ OR exp Telenursing/ OR exp Personal Digital Assistant/ OR exp Wireless communication/ OR exp Technology/ OR exp Digital Technology/ OR exp Educational Technology/ OR exp Communication Technology/ OR exp Telephone/ OR exp Videoconferencing/ OR exp E-Mail/ |
| #2 | ((digital ADJ intervention) OR (digital ADJ treatment) OR (digital ADJ therap*)).ti,ab,kf,kw. |
| #3 | ((technology-based ADJ intervention) OR (technology-based ADJ treatment) OR (technology-based ADJ therap*)).ti,ab,kf,kw. |
| #4 | ((technology-mediated ADJ intervention) OR (technology-mediated ADJ treatment) OR (technology-mediated ADJ therap*)).ti,ab,kf,kw. |
| #5 | ((technology-delivered ADJ intervention) OR (technology-delivered ADJ treatment) OR (technology-delivered ADJ therap*)).ti,ab,kf,kw. |
| #6 | ((technology-enhanced ADJ intervention) OR (technology-enhanced ADJ treatment) OR (technology-enhanced ADJ therap*)).ti,ab,kf,kw. |
| #7 | (JITAI* or "just-in-time-adaptive-intervention*").ti,ab,kf,kw. |
| #8 | ((electronic* OR medication*) ADJ3 (reminder* OR monitor* or record* OR system* OR device*)).ti,ab,kf,kw. |
| #9 | (reminder ADJ3 (text* or system* or messag*)).ti,ab,kf,kw. |
| #10 | (alert*).ti,ab,kf,kw. |
| #11 | (sms OR mms).ti,ab,kf,kw. |
| #12 | ("social media" OR WhatsApp).ti,ab,kf,kw. |
| #13 | (video* OR television OR radio OR media* OR multimedia OR “multi‐media”).ti,ab,kf,kw. |
| #14 | (texting).ti,ab,kf,kw. |
| #15 | ((text* OR short*) ADJ3 messag*).ti,ab,kf,kw. |
| #16 | (telehealth* OR “tele‐health*” OR telecare* OR “tele‐care*” OR telemedicine* OR “tele‐medicine*” OR telepharmacy OR “tele‐pharmacy”).ti,ab,kf,kw. |
| #17 | (mhealth or "m‐health" or "m health" or "mobile health").ti,ab,kf,kw. |
| #18 | ("e‐health" or ehealth or "e health").ti,ab,kf,kw. |
| #19 | (telenurs* or "tele‐nurs*" or "tele‐homecare" or telehomecare or teleconsultation or "tele‐consultation").ti,ab,kf,kw. |
| #20 | ((remote* or distant or distance) ADJ (consult* or monitor* or care or treat* or therap*)).ti,ab,kf,kw. |
| #21 | ((mobile* or digital*) ADJ health*).ti,ab,kf,kw. |
| #22 | (online* OR web* OR browser OR portal OR internet* OR virtual*).ti,ab,kf,kw. |
| #23 | (personal* ADJ1 digital* ADJ1 assistant*).ti,ab,kf,kw. |
| #24 | (app* ADJ3 (smartphone* or "smart‐phone" or mobile* or phone* or tablet* or computer*)).ti,ab,kf,kw. |
| #25 | (telephone or phone*).ti,ab,kf,kw. |
| #26 | (email or "e‐mail" or electronic mail).ti,ab,kf,kw. |
| #27 | (interactive* or telecommunication*).ti,ab,kf,kw. |
| #28 | (wireless* or bluetooth*).ti,ab,kf,kw. |
| #29 | #1 OR #2 OR #3 OR #4 OR #5 OR #6 OR #7 OR #8 OR #9 OR #10 OR #11 OR #12 OR #13 OR #14 OR #15 OR #16 OR #17 OR #18 OR #19 OR #20 OR #21 OR #22 OR #23 OR #24 OR #25 OR #26 OR #27 OR #28 |
|  | **ALCOHOL USE** |
| #30 | Exp alcohol/ OR exp alcoholism/ OR exp drinking behavior/ OR exp drinking/ OR exp binge drinking/ OR exp heavy drinking/ OR exp alcohol consumption/ OR exp alcohol abuse/ |
| #31 | (alcohol* OR alcohol-related OR drink*).ti,ab,kf,kw. |
| #32 | 30 OR 31 |
| #33 | (addict* OR abus* OR misus* OR disorder* OR “mis-use” OR dependen* OR "use disorder*" OR related disorder* OR risk* OR hazardous* OR problem* OR binge OR heavy OR harmful).ti,ab,kf,kw. |
| #34 | 32 AND 33 |
|  | **REGION** |
| #35 | (afghan* or africa* or albania* or algeria* or angola* or antigua* or barbuda* or argentin* or armenia* or aruba* or azerbaijan* or bahrain* or bangladesh* or bengal* or bangal* or barbados* or barbadian* or bajan or bajans or belarus* or belorus* or byelarus* or byelorus* or belize* or benin* or dahomey or bhutan* or bolivia* or bosnia* or herzegovin* or botswan* or batswan* or bechuanaland* or brazil* or brasil* or bulgaria* or burkina* or burkinese* or upper volta* or burundi* or urundi* or cabo verde* or cape verde* or cambodia* or kampuchea* or khmer* or cameroon* or cameroun* or ubangi shari* or chad* or chile* or china* or chinese or colombia* or comoro* or comore* or comorian* or mayotte* or congo* or zaire* or costa rica* or "cote d'ivoir*" or "cote d' ivoir*" or cote divoir* or cote d ivoir* or ivory coast* or ivorian* or croatia* or cuba or cuban or cubans or "cuba's" or cyprus* or cypriot* or czech* or djibouti* or french somaliland* or dominica* or ecuador* or egypt* or united arab republic* or el salvador* or salvadoran* or guinea* or equatoguinea* or eritrea* or estonia* or eswatini* or swaziland* or swazi* or swati* or ethiopia* or fiji* or gabon* or gabonese* or gabonaise* or gambia* or ((georgia or georgian or georgians) not (atlanta or california or florida)) or ghana* or gibraltar* or greece* or greek* or grecian* or grenada* or grenadian* or guam* or guatemala* or guyana* or guiana* or guyanese* or haiti* or hispaniola* or hondura* or hungary* or hungarian* or india* or indonesia* or iran* or iraq* or isle of man* or jamaica* or jordan* or kazakh* or kenya* or karabati* or korea* or kosovo* or kosova* or kyrgyz* or kirgiz* or kirghiz* or laos or lao or laotian* or latvia* or lebanon* or lebanese* or lesotho* or lesothan* or lesothonian* or basutoland* or mosotho* or basotho* or liberia* or libya* or jamahiriya* or lithuania* or macedonia* or madagasca* or malagasy* or malawi* or nyasaland* or malaysia* or malay* federation or maldives* or maldivian* or indian ocean or mali or malian* or "mali's" or malta or maltese* or "malta's" or micronesia* or marshallese* or kiribati* or marshall island* or nauru or nauran or nauruans or "naurian's" or mariana or marianas or palau or paluan* or tuvalu* or mauritania* or mauritan* or mauritius* or mexico* or mexican* or moldova* or moldovia* or mongol* or montenegr* or morocco* or moroccan* or ifni or mozambique* or mozambican* or myanmar* or burma* or burmese or namibia* or nepal* or new caledonia* or netherlands antill* or nicaragua* or niger* or oman or omani or omanis or "oman's" or pakistan* or palestin* or gaza* or west bank* or panama* or paraguay* or peru or peruvian* or "peru's" or philippine* or philipine* or phillipine* or phillippine* or filipino* or filipina* or poland* or polish or pole or poles or portugal* or portuguese or puerto ric* or romania* or russia* or ussr* or soviet* or rwanda* or rwandese or ruanda* or ruandese or samoa* or navigator island* or pacific island* or polynesia* or "sao tome and principe*" or sao tomean* or santomean* or saudi arabia* or saudi or saudis or senegal* or serbia* or seychell* or sierra leone* or slovak* or sloven* or melanesia* or solomon island* or norfolk island* or somali* or sri lanka* or ceylon* or "saint kitts and nevis*" or "st kitts and nevis*" or kittian* or nevisian* or saint lucia* or st lucia* or saint vincent* or st vincent* or vincentian* or grenadine* or sudan* or surinam* or syria* or tajik* or tadjik* or tadzhik* or tanzania* or tanganyika* or thai* or timor leste* or east timor* or timorese* or togo or togoles* or "togo's" or tonga* or trinidad* or tobago* or tunisia* or turkiy* or turkey* or turk or turks or turkish or turkmen* or uganda* or ukrain* or uruguay* or uzbek* or vanuatu* or new hebrides* or venezuela* or vietnam* or viet nam* or yemen* or yugoslav* or zambia* or zimbabwe* or rhodesia* or arab* countr* or middle east* or global south or sahara* or subsahara* or magreb* or maghrib* or west indies* or caribbean* or central america* or latin america* or south america* or central asia* or north asia* or northern asia* or southeastern asia* or south eastern asia* or southeast asia* or south east asia* or west asia* or western asia* or east europe* or eastern europe* or developing countr* or developing nation* or developing population* or developing world or less developed countr* or less developed nation* or less developed world or lesser developed countr* or lesser developed nation* or lesser developed world or under developed countr* or under developed nation* or under developed world or underdeveloped countr* or underdeveloped nation* or underdeveloped world or middle income countr* or middle income nation* or middle income population* or low income countr* or low income nation* or low income population* or lower income countr* or lower income nation* or lower income population* or underserved countr* or underserved nation* or underserved population* or under served population* or under served nation* or under served population* or deprived countr* or deprived population* or high burden countr* or high burden nation* or countdown countr* or countdown nation* or poor countr* or poor nation* or poor population* or poor world or poorer countr* or poorer nation* or poorer population* or poorer world or developing econom* or less developed econom* or underdeveloped econom* or under developed econom* or middle income econom* or low income econom* or lower income econom* or low gdp or low gnp or low gross domestic or low gross national or lower gdp or lower gnp or lower gross domestic or lower gross national or lmic or lmics or third world or lami countr* or transitional countr* or emerging econom* or emerging nation*).ti,ab,hw,kf. |
| #36 | 29 AND 34 AND 35 |
|  | **NOT ANIMALS** |
| #37 | exp animals/ not humans.sh. |
| #38 | 36 NOT 37 |

**Medical Subject Headings (MeSH terms)**

|  | **Digital interventions (#1)** | **Alcohol use (#30)** |
| --- | --- | --- |
| **MEDLINE** | exp Web Browser/ OR exp Computer Communication Networks/ OR exp Online Systems/ OR exp Internet/ OR exp Cell Phone/ OR exp MP3 Player/ OR exp Computer Systems/ OR exp Microcomputers/ OR exp Mobile Applications/ OR exp Communications Media/ OR exp Social Media/ OR exp Reminder Systems/ OR exp Telemedicine/ OR exp Text Messaging/ OR exp Telenursing/ OR exp Computers, Handheld/ OR exp Computers/ OR exp Wireless Technology/ OR exp Technology/ OR exp Telephone/ OR exp Telecommunications/ OR exp Videoconferencing/ OR exp Electronic Mail/ | Exp alcohol/ OR exp Alcoholism/ OR exp Alcohol Drinking/ OR exp Drinking/ OR exp Binge Drinking/ OR exp Drinking Behavior/ OR exp Alcohol-Related Disorders/ |
| **Global Health** | exp Web sites/ OR exp Internet/ OR exp mobile telephones/ OR exp Computers/ OR exp Microcomputers/ OR exp Mobile Applications/ OR exp Social Media/ OR exp Telemedicine/ OR exp Text Messaging/ OR exp Technology/ OR exp digital Technology/ OR exp educational technology/ OR exp Telephones/ OR exp Telecommunications/ | exp Alcoholism/ OR exp Alcohol intake/ OR exp Alcoholic beverages/ OR exp Drinking/ |
| **PsycInfo** | exp Internet/ OR exp Computer applications/ OR exp Social Networks/ OR exp Online Social Networks/ OR exp Computer Mediated Communication/ OR exp Mobile Phones/ OR exp Computers/ OR exp Microcomputers/ OR exp Mobile Applications/ OR exp Communications Media/ OR exp Social Media/ OR exp Telemedicine/ OR exp Text Messaging/ OR exp Human Computer Interaction/ OR exp Wireless Technologies/ OR exp Technology/ OR exp Telephone systems/ OR exp Telecommunications media/ OR exp “Information and Communication Technology”/ OR exp Teleconferencing/ OR exp videoconferencing/ OR exp Online therapy/ | exp Alcoholism/ OR exp Alcohol Abuse/ OR exp “Alcohol Use”/ OR exp “Alcohol Use Disorder”/ OR exp Binge Drinking/ OR exp Drinking Behavior/ OR exp Alcohol Treatment/ OR exp Alcohol Treatment/ OR exp Alcohol Withdrawal/ |
